# Fibroblast feeder layer supports adipogenic differentiation of human adipose stromal/progenitor cells

**DOI:** 10.1080/21623945.2019.1608751

**Published:** 2019-04-29

**Authors:** Asim Ejaz, Florian M Hatzmann, Sarina Hammerle, Heike Ritthammer, Monika Mattesich, Marit Zwierzina, Petra Waldegger, Werner Zwerschke

**Affiliations:** a Division of Cell Metabolism and Differentiation Research, Research Institute for Biomedical Aging Research, University of Innsbruck, Innsbruck, Austria; b Department of Plastic and Reconstructive Surgery, Innsbruck Medical University, Innsbruck, Austria; c Center for Molecular Biosciences Innsbruck (CMBI), University of Innsbruck, Innsbruck, Austria

**Keywords:** Aging, adipose stem cells, adipogenesis, fibroblast feeder layer, obesity

## Abstract

Adipose stromal/progenitor cells (ASCs) can differentiate into adipocytes in the course of adipogenesis. This process is governed by systemic factors and signals of the adipose stem cell niche. ASCs isolated from fat tissues and amplified* in vitro* provide an essential and reliable model system to study adipogenesis. However, current cell culture models routinely grow ASCs on plastic surfaces largely missing niche parameters. In the present communication, we employed human foreskin fibroblasts (HFFs) monolayers as feeder cells for ASCs, which were isolated from human subcutaneous white adipose tissue and amplified *in vitro*. We found that PPARγ2 and several adipocyte markers were significantly higher expressed in differentiated ASCs growing on feeder layers relative to plastic dishes. Moreover, a significant higher number of adipocytes was generated from ASCs cultured on feeder layer and these adipocytes contained larger fat droplets. Insulin strongly stimulated glucose uptake into adipocytes produced on feeder layer suggesting that these cells show characteristic metabolic features of fat cells.  Finally, we show that the HFF feeder layer allows adipogenic differentiation of low-density-seeded ASCs. In conclusion, we demonstrate that the HFF feeder layer increases adipocyte differentiation of ASCs and allows differentiation of low density seeded progenitor cells  into functional adipocytes.

## Introduction

Obesity is an important risk factor contributing to the overall burden of disease [1]. White adipose tissue (WAT) as major triglyceride storage and metabolic regulator buffers nutrient availability and demand by storing excess calories and delivering free fatty acids during fasting [2,3]. It is a highly plastic organ, and its enlargement plays an important part in obesity and its associated pathologies [4]. Growth of WAT is a result of an increase in number and size of adipocytes due to their inherent renewal and expansion capacity [5]. Adipocytes differentiate from adipose stromal/progenitor cells (ASCs). Human ASCs in subcutaneous (s)WAT are increasingly well determined and can be characterized by the cell surface marker combination DLK1^−^/CD34^+^/CD90^+^/CD146^−^/CD31^−^/CD45^−^ [6–9]. These cells are thought to be a subpopulation of a heterogeneous population of ASCs localized in the stroma surrounding small vessels [6–8,10–12], which constitutes an important stem cell niche in sWAT [13,14]. Adipocyte differentiation (adipogenesis) is a multistep process governed by a complex signal transducing network including endocrine pathways, such as insulin-signalling and less well-understood determinants from the local environment [4]. Niche cells in general contribute nurturing and supporting soluble and insoluble factors and proteins that assemble to form a three‑dimensional matrix network, the extracellular matrix (ECM) [6–8,10–14]. ASCs are localized in vicinity to niche cells (stromal cells, endothelial cells, adipocytes, others) and are embedded in the ECM. The composition [15–18] and rigidity of the ECM [19–24] are among the factors responsible for regulating ASC commitment. In addition, cell confluence [25], shape [26], and adhesion [27] further contribute signals involved in regulating ASC commitment. These structural features impose shear, tension, and stretching forces on ASCs that activate mechano-transduction pathways [28], which are important to direct the three-dimensional development of adipose tissue [4,19,22,28,29]. Moreover, a recent study detected specialized regulatory stromal cells in the niche that influence adipogenesis [30]. Altogether, endocrine, paracrine, and autocrine-soluble and -insoluble factors and proteins in the niche trigger ASC lineage commitment and eventually activate or repress a transcriptional cascade of adipogenic factors necessary for terminal differentiation [4,31,32]. In the centre of this cascade are the adipogenic key factors PPAR-γ2 and C/EBPα, which drive terminal adipocyte differentiation [4].

ASCs are isolated from the stromal vascular fraction (SVF) of disaggregated adipose tissue by their property to adhere to plastic surfaces [33]. *In vitro* cell culture models routinely expand ASCs on plastic dishes largely missing niche parameters, such as neighbouring cells and ECM. This leads to changes in the surface marker potpourri of ASCs [34] and limits their proliferation and adipocyte differentiation capacity [35], in part due to missing signals from cell surface protein of surrounding cells and ECM [36,37] but also due to lack of soluble nurturing and steering niche factors [38]. One established model system to mimic and compensate the missing stem cell niche *in vitro* is the use of feeder cells monolayer [38]. In the present study, we employed a feeder layer system consisting of a monolayer of human foreskin fibroblasts (HFFs) to test whether these cells provide a surrogate model for mimicking the ASC cellular niche environment. The HFF monolayer was used to provide feeder cells for human ASCs isolated from abdominal sWAT and amplified *in vitro* on plastic dishes to passage 6 (P6). We found a strong increase in adipogenic differentiation capacity by employing the feeder layer system.

## Material and methods

### Donors

Human sWAT samples were taken from persons undergoing routine abdominoplasty at the Institute for Plastic and Reconstructive Surgery at the Medical University of Innsbruck, Innsbruck, Austria [33]. The patients gave their informed written consent, and the study had been approved by the ethical committee of Innsbruck Medical University, Austria, according to the Declaration of Helsinki. All sWAT samples were obtained from the lower abdomen. The clinical and anthropometric parameters are indicated in Table 1.

**Table 1.** Clinical and anthropometric parameters of the donors.

Donor	Sex	Age (years)	Height (cm)	Weight (kg)	BMI (kg/m^2^)
1	f	28	158	54	21.63
2	m	n.a.	n.a.	n.a.	n.a.
3	f	33	168	68	24.09
4	f	39	165	73	26.81
5	m	55	n.a.	100	n.a.
6	f	46	158	55	22.03
7	f	35	167	62	22.23
8	f	29	172	68	22.99

Human sWAT samples were taken from the lower abdomen from healthy Caucasian donors undergoing routine abdominoplasty at the Institute for Plastic and Reconstructive Surgery at the Medical University of Innsbruck, Austria.

f, female; m, male; n.a., not available.

#### Isolation of human ASCs

The adipose precursor cells were isolated essentially as described [33]. Adipose tissue biopsies after surgery procedures were transferred to lab in sterile serum-free ASC medium (Dulbecco’s modified Eagle’s medium [DMEM]/F-12 medium (1:1) with HEPES and L-glutamine (Sigma, Vienna, Austria), supplemented with 33 μM biotin (Sigma), 17 μM pantothenate (Sigma), and 12.5 μM/ml gentamicin (Sigma) and kept at 4°C for 1–3 h before sterile processing in a Class II Biosafety Cabinet (ESCO). Tissue was rinsed thrice with Dulbecco's phosphate buffered saline (PBS; Sigma, #D8537) followed by removal of fibrous material and blood vessels by dissection. The tissue was cut into pieces (1–2 mg) and digested in digestion buffer (PBS containing 200 U/ml collagenase (CLS Type I, Worthington Biochemical Corp., Lakewood, NJ, USA) and 2% w/v BSA (Roth, Germany)) under stirring for 60 min at 37°C ; 1 mg adipose tissue/3 ml digestion buffer. The dispersed tissue was centrifuged for 10 min at 200 *g* at room temperature. The floating adipocytes were aspirated and the pelleted cells of the SVF were suspended in erythrocyte lysis buffer (0.155 M NH4CI, 5.7 mM K2HPO4, 0.1 mM EDTA, pH 7.3) and incubated for 10 min at room temperature. To remove tissue debris, the cell suspension was filtered through a nylon mesh (pore size 100 μm, BD, Wien, Austria). After another centrifugation step (10 min at 200 *g*) the pelleted SVF was suspended in ASC medium supplemented with 10% fetal bovine serum(FBS; Sigma, #F7524), and filtered through a 35-μm mesh to remove residual cell aggregates. SVF cells were seeded at a density of 70,000/cm^2^ into 6-well cell culture dishes. After 16 h of cell attachment, cells were washed with ASC medium to remove non-adhering cells. Thereafter the confluent cells were maintained for 6 d in ASC medium without FBS. Medium was changed every second day. This led to the elimination of all non-adherent cells. The remaining cell population was referred to as passage 1 of the ASC fraction, which was used for further studies. This cell population contains an enriched population of ASCs.

#### Cultivation of ASCs

ASCs were expanded essentially as described [33]. The cells were washed with PBS and trypsinized using 0.05% trypsin-EDTA 1x solution (Sigma). Trypsin was inactivated by addition of ASC medium plus 10% FBS and removed by centrifugation at 300 *g* for 5 min and the cells seeded in a density of 5000 cells/cm^2^ in ASC medium plus 10% FBS and maintained at 37°C with 5% CO_2_. Sixteen hours later, the medium was replaced by PM4 medium (ASC medium containing 2.5% FBS, 10 ng/ml EGF (Immuno Tools Friesoythe, Germany), 1 ng/ml bFGF (Immuno Tools, Germany), 500 ng/ml insulin (Sigma). ASC were passaged at a ratio of 1: 2, medium was changed every third day and the cells were grown to 70% confluence before splitting. Population doublings (PDL) were calculated using the following equation: 1 PDL = Log_10_ (*N*/*N*°) × 3.33 (*N* = number of cells at the end of a passage, *N*° = number of cells that were seeded at the beginning of a passage) [39]. For storage cells were pelleted as described above, diluted in cryomedium (DMEM/F-12 medium (1:1) (1x) with HEPES and L-glutamine (Sigma), with 20% FBS and 7.5% DMSO), at a density of 10^6^ cells/ml, slowly brought to – 80°C and then stored in frozen nitrogen. After thawing, cryomedium was immediately removed by centrifugation. ASCs cultivated to P6 were used in this study.

#### Cultivation of HFFs

HFFs were a gift from Pidder Jansen-Dürr (Research Institute for Biomedical Aging Research, University of Innsbruck, Innsbruck, Austria). The cells were cultivated as described [39]. Briefly, 30,000 cells/cm^2^ were seeded in 6-well cell culture dishes in DMEM (Sigma) supplemented with 10% FCS. Cells were grown to confluency and used as feeder layer for ASCs or used alone as control cells.

#### Adipogenic differentiation

For the induction of adipogenesis, the ASC were seeded at a density of 50,000 cells/cm^2^ either directly on the plastic surface of cell culture dishes or on a confluent layer of HFFs in 6-well cell culture dishes in ACS medium supplemented with 10% FCS. After a resting period of 24 h in ASC medium, adipogenesis was induced using differentiation medium, composed of 0.2 μM insulin (Roche, Vienna, Austria), 0.5 mM 1-methyl-3-isobutylxanthine (IBMX) (Sigma), 0.25 μM dexamethasone (Sigma), 2.5% FBS, and 10 μg/ml transferrin (Sigma) in ASC medium. After d 3 of differentiation, the medium was changed and the cells were cultivated in differentiation medium without IBMX.

#### Quantification of the number of adipogenic differentiated cells in ASC low-density seedings

We seeded 1000 ASCs per well over feeder layer and induced differentiation. Wells were stained with Oil red O and number of positive cells were counted.

#### Oil-Red O staining

For visualization of lipid droplets, cells were fixed with 4% paraformaldehyde in PBS for 1 h and stained with 0.3% Oil-Red O (Sigma) in isopropanol/water (60:40) for 1 h. Final washing was carried out twice with distilled water. For quantification of absorbed Oil-Red O, the stain was eluted with isopropanol (Sigma) and optical density was measured at 518 nm.

#### LipidTox-green staining

On d 14 post induction of adipogenesis, cells were fixed using 4% PFA, washed twice with PBS and subsequently stained with LipidTox Green (Invitrogen) 1:200 and DAPI 1:200 for 1 h at room temperature. Images were acquired using the fluorescent microscope CV1000 and analysed with the CV1000 software.

#### Quantitative RT-PCR gene expression analysis

Total RNA was isolated with the RNeasy Micro Kit (Qiagen, Hilden, Germany), and cDNA synthesis was performed with the First Strand cDNA Synthesis Kit (Fermentas, St. Leon-Rot, Germany). Quantitative expression analysis was performed using the LightCycler® 480 Real-Time PCR System (Roche). The mRNA quantification was performed using *β-actin* for normalization. The efficiencies of the primers used were calculated. Data for each gene transcript were normalized by calculating the difference (∆Ct) from the Ct-housekeeping and Ct-Target genes. The relative increase or decrease in expression was calculated by comparing the reference gene with target gene calculated by comparing the reference gene with the target gene (∆∆Ct) and using the formula for relative expression (= ^2∆∆Ct^). The sequences of the primers within the sequence are indicated in Table 2.

**Table 2.** The primer sequences used for qRT-PCR analysis are indicated.

Gene	Direction	Sequence
*β-Actin*	Forward Reverse	5`AGA AAA TCT GGC ACC ACA CC 3` 5`AGA GGC GTA CAG GGA TAG CA 3`
*FABP4*	Forward Reverse	5`CAG TGT GAA TGG GGA TGT GA 3` 5`CGT GGA AGT GAC GCC TTT 3`
*PPARγ2*	Forward Reverse	5`ATG GGT GAA ACT CTG GGA GA 3` 5`TGG AAT GTC TTC GTA ATG TGG A 3`
*Perilipin*	Forward Reverse	5`GAC AAC GTG GTG GAC ACA GT 3` 5`CTG GTG GGT TGT CGA TGT C 3`
*Adiponectin*	Forward Reverse	5`CCT GGT GAG AAG GGT GAG AA 3` 5`GTA AAG CGA ATG GGC ATG TT 3`

#### Western blot analysis

Western blot analysis was performed essentially as described [40]. The method used to normalize the protein levels was “Pierce BCA Protein Assay Kit” (#23227BCA). Cell lysates (15 μg total protein per lane) were prepared in sodium dodecyl sulphate (SDS) sample buffer and separated by SDS-PAGE and blotted on polyvinylidene difluoride membranes. Following antibodies were used: Mouse anti-human β-actin (dilution 1: 100.000) (Sigma, cat # A5441) and perilipin (dilution 1: 500) (Cell Signaling, USA), anti-mouse IgG HRP conjugate (Promega, Mannheim #W420B) (dilution 1: 5.000), rabbit anti-rat IgG HRP (Dako Cytomation, Hamburg, #P0450) (dilution 1: 10.000). ImageJ software was used for densitometric analyses.

#### Glucose uptake assay

The same number of cells were cultured in 6-well plates either directly on plastic or on a feeder layer. On d 14 post induction of adipogenesis, the medium was changed to ASC medium without insulin. After 1 d incubation in insulin-free medium, the medium was changed to insulin containing differentiation medium without IBMX. After 1-h incubation at 37°C, 100 µM of the fluorescently labelled glucose analogue 2-NBDG (Invitrogen) was added. Ten minutes later, the reaction was stopped by washing twice with PBS. The cells were kept in PBS+1% BSA for analysis. Images were acquired using Nikon Eclipse TE300 fluorescent microscope and analysed by NIS-elements software and ImageJ.

#### Statistical analysis

Statistical analyses was performed in GraphPad Prism (GraphPad Software Inc., La Jolla, CA, USA). The significance of difference between means was assessed by Student’s *t*-test or analysis of variance. Error bars are represented as the mean ± SEM.

## Results

**Figure 1.**
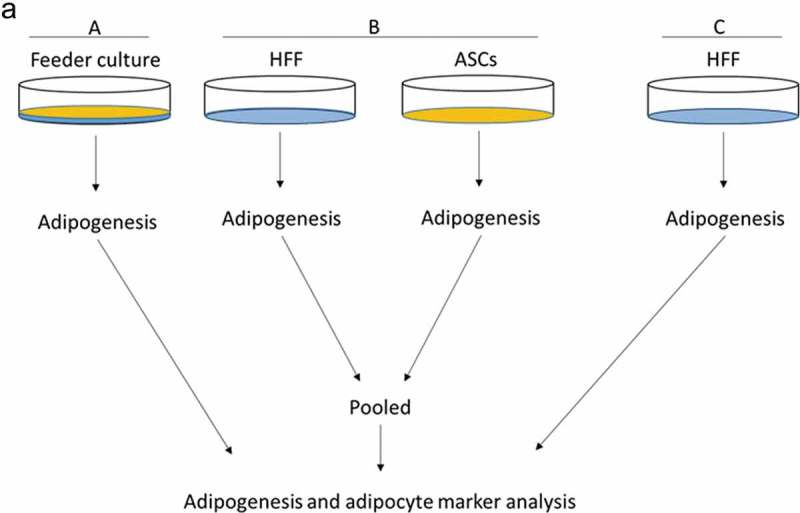
Experimental scheme. (a) HFFs were grown as feeder monolayer (blue) in co-culture with ASCs (yellow) (feeder culture). (b) Both HFFs and human ASCs were cultivated independently in pure culture on plastic cell culture dishes (HFF + ASCs). (c) HFFs were grown in pure culture on cell culture dishes (HFF). Afterwards, adipogenesis was induced by hormone cocktail and efficiency of adipogenic differentiation was monitored using adipogenesis and adipocyte marker.

**Figure 2.**
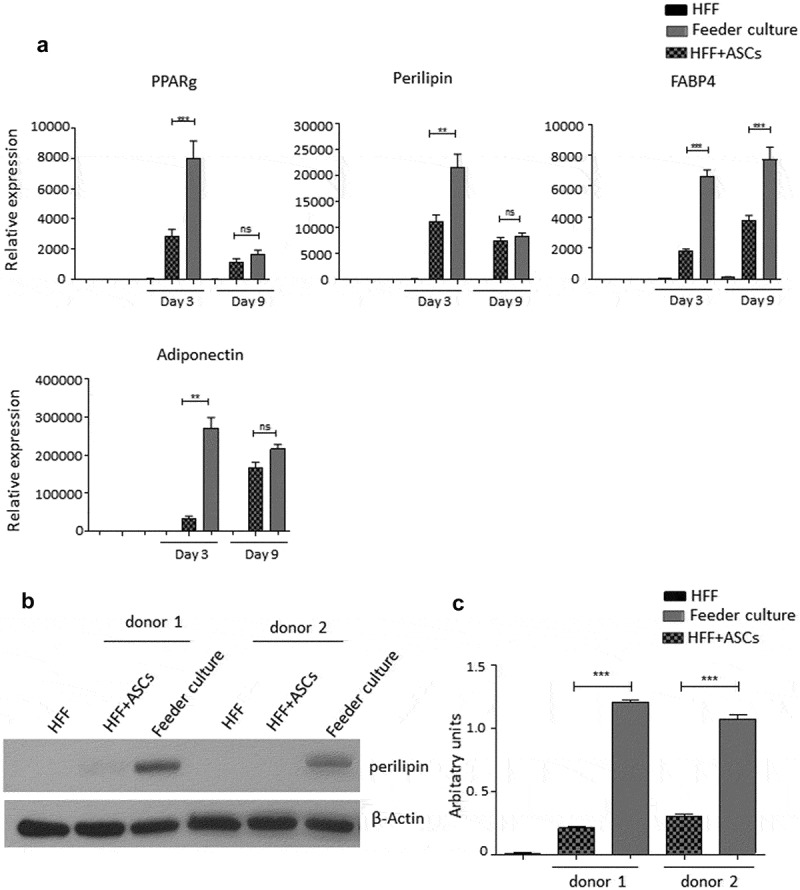
Adipogenic differentiation of human ASCs is enhanced upon feeder with HFFs. Cells were grown according to the scheme shown in Figure 1. Adipogenesis was induced, lysates were prepared and PPARγ2, perilipin, FABP4 and adiponectin mRNA expression was estimated at d 3 and 9 post differentiation. Gene expression before differentiation induction was taken as 1, and fold changes in gene expression were calculated. Graphs are representative of 2 biological repeats (a). Perilipin protein expression was analysed by Western blot, *β-actin* was used as input control (b). Perilipin and *β-actin* band intensities were quantified using ImageJ software and ratio of perilipin to *β-actin* was plotted as arbitrary units (c). All error bars represent the means ± SEM. p* *p* < 0.05, **p < 0.001 and ***p < .0001. Analysis of variance (ANOVA) is applied for (a) and (c).

To mimic the effects of the stem cell niche microenvironment on adipogenic differentiation of human ASCs in culture, we employed a monolayer of density-arrested HFF as a feeder basement. ASCs were amplified on plastic cell culture dishes until P6. Afterwards, the following experimental strategy was employed (Figure 1). ASCs were seeded as a confluent monolayer on the top of a confluent HFF monolayer and subsequently incubated with adipogenic differentiation medium (Figure 1(a)). Both the HFFs and the ASCs were also separately cultivated as monolayers on plastic cell culture dishes and treated with adipogenic differentiation medium. Afterwards, both cell types were pooled to compensate for the HFF cells in the feeder co-culture experiment, which could potentially interfere in the molecular analysis of adipogenesis markers (Figure 1(b)). As an additional control HFFs were grown to confluence in pure culture on plastic cell culture dishes and treated with adipogenic differentiation medium (Figure 1(c)). We detected significantly higher mRNA expression level of the adipogenic key regulator PPARγ2 at d 3 post induction of adipogenesis in feeder culture (Figure 2(a)). The mRNA expression level of the adipocyte marker FABP4 was significantly higher at d 3 and 9 post induction of adipocyte differentiation in the feeder culture (Figure 2(a)). The mRNA expression level of perilipin and adiponectin, two additional adipocyte markers, was significantly higher at day 3 post induction of adipocyte differentiation in feeder culture (Figure 2(a)). Moreover, Western blot analyses demonstrated a significant higher perilipin protein level in premature adipocytes at d 9 post differentiation induction in the feeder culture system (Figure 2(b,c)). Next, we analysed the efficiency of adipogenesis at day 14 post induction of differentiation by imaging lipid droplet containing premature adipocytes using light microscopy (Figure 3(a,b)) and after fixing and staining of lipids by Oil-Red O (Figure 3(c)). We observed a considerable higher number of adipocytes derived from feeder layer-cultured ASCs relative to ASCs cultivated on plastic dishes (Figure 3(c,d)). Moreover, we detected stronger differentiated adipocytes in feeder culture relative to plastic culture as depicted by the larger volume of the Oil-Red O-stained fat droplets (Figure 3(e)). Quantification of the Oil-Red O dye absorbed by the given differentiated cells underscored that the feeder culture led to significantly stronger adipocyte differentiation (Figure 3(f)). The supporting effect of feeder layer culture on adipogenic differentiation of ASCs is also reflected by whole well photograph after staining with Oil-Red O (Figure 3(g)). Staining by the neutral lipid stain LipidTox further corroborated our findings that adherence to HFF feeder layers better supports adipogenic differentiation of ASCs than adherence to plastic surfaces (Figure 3(h)). To provide additional evidence that adipocytes produced on fibroblast monolayers possess phenotypic characteristics similar to adipocytes isolated directly from WAT, we measured whether HFF monolayer-produced adipocytes take up the fluorescently labelled glucose analogue 2-NBDG in response to insulin. To do this, ASCs were seeded to density on HHF feeder layers and on plastic dishes and differentiated into adipocytes. HFF alone served as control. As shown in Figure 3(i), insulin strongly stimulated the uptake of 2-NBDG into the adipocytes that were produced on the feeder layer. The 2-NBDG uptake was higher in HFF monolayer-produced adipocytes than in adipocytes generated on plastic dishes. These data indicate that HFF monolayer-produced adipocytes show characteristic metabolic features of adipocytes.

**Figure 3.**
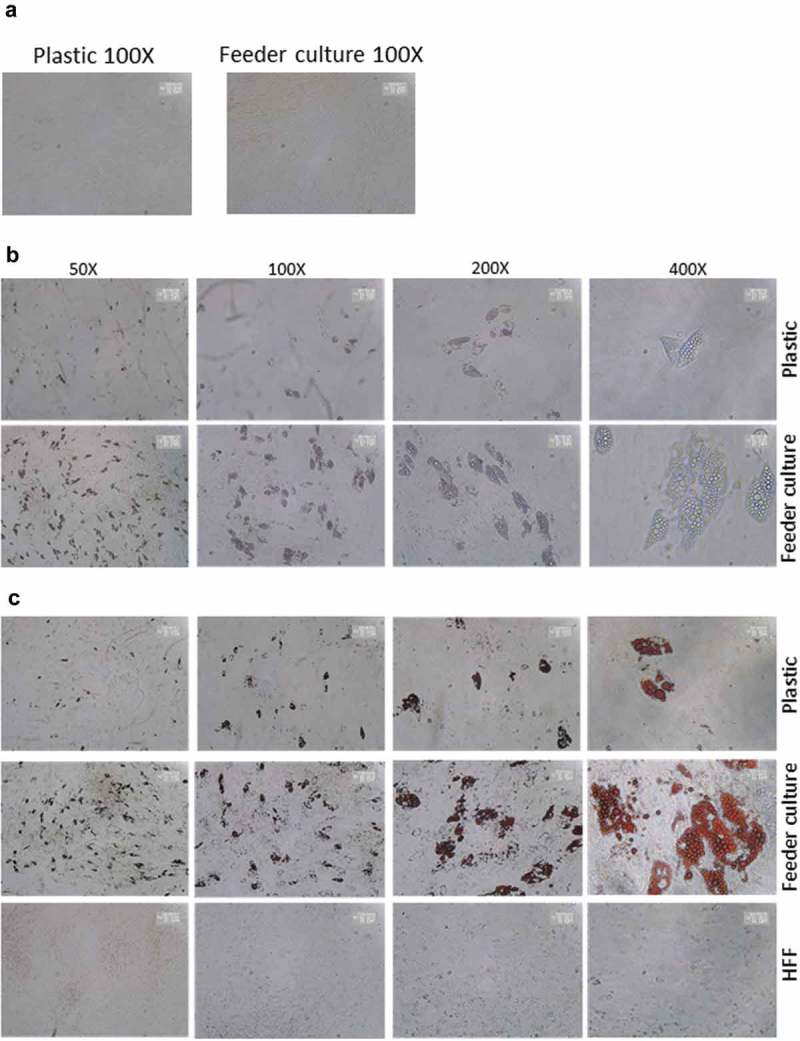
HFF feeder culture increases adipocyte formation. (a) Light microscopic images of ASCs in pure culture (left) and ASCs in co-culture with HFF feeder (right) are shown. (b) Adipocyte differentiation was induced by hormone cocktail and ASCs in pure culture on plastic dishes and co-culture with HFF feeder were imaged using a light microscope at d 14 post induction of differentiation to estimate the formation of lipid droplets. Representative images from three biological repeats are shown. (c) Accumulation of lipids at d 14 post differentiation was confirmed using Oil-Red-O staining. Representative images from three biological repeats are shown. (d) The number of Oil-Red-O positive cells formed in pure culture on plastic dishes and in feeder culture is indicated. Three biological repeats were employed. (e) The size of fat droplets in formed adipocytes in pure culture on plastic dishes and in feeder culture was measured using ImageJ software and plotted as arbitrary units. Cells from three donors were employed. (f) Oil-Red-O uptake by formed adipocytes in pure culture and feeder culture was quantified by eluting the stain in isopropanol and measuring the absorbance at 518 nm. Graphs are representative of two biological repeats. (g) Feeder culture leads to an enhanced adipogenic differentiation as depicted by whole well image after staining with Oil-Red-O. Images are representative of two biological repeats. (h) D 14 post induction of adipocyte differentiation in pure culture on plastic dishes and in combination with HFFs in feeder culture cells were fixed and stained with LipidTox (green) while nuclei are stained with DAPI (blue), 400× magnification. *n* = 3. (i) Insulin-stimulated glucose uptake in human ASCs grown on plastic and feeder layer. (Left panel) ASCs were cultured either directly on plastic or on feeder layer until d 14 post induction of differentiation. HFFs alone served as control. After incubation in insulin-free medium for 1 d, cells were stimulated with medium containing insulin. One hour later, 2-NBDG was added for 10 min. The reaction was stopped by washing with PBS, and 2-NBDG uptake was analysed by fluorescence microscopy. *N* = 3 donors; representative images from one donor are shown. (Right panel) Fluorescence was measured using ImageJ, the mean fluorescence from HFFs was subtracted as background. For quantification, five images per donor were used. All error bars represent the mean ± SEM. *p < 0.05, ** *p* < 0.001 and ***p < .0001. Analysis of variance (ANOVA) is applied for (F), while Student’s *t*-test is applied to (d), (e) and (i).

**Figure 3.**
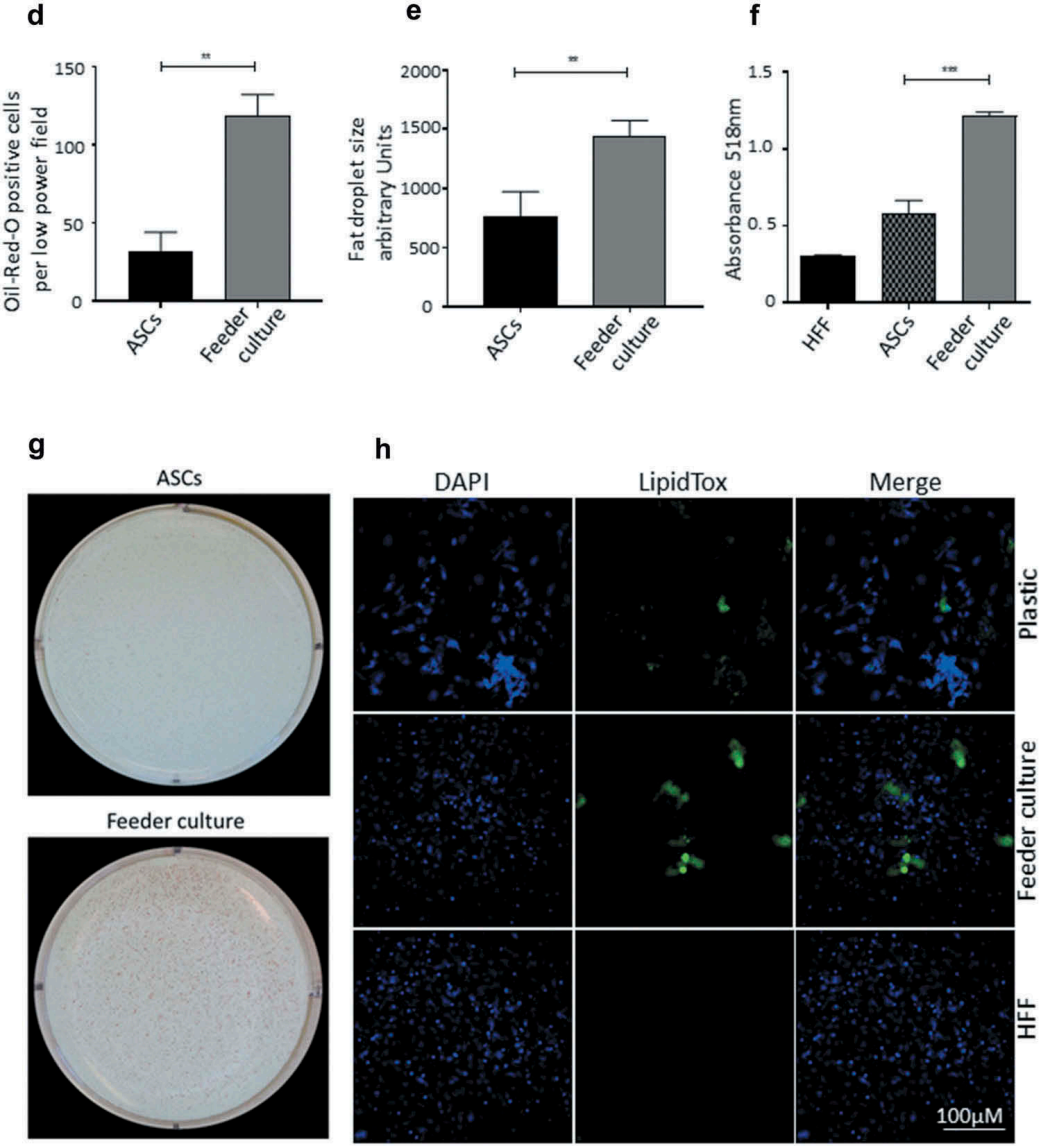
(Continued).

**Figure 3.**
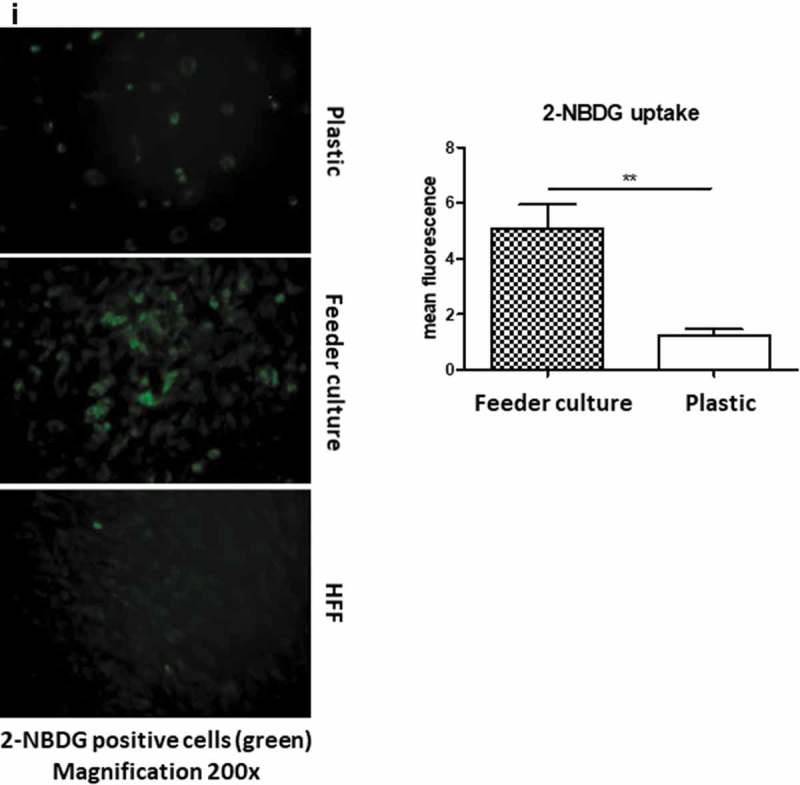
(Continued).

SVFs derived from human sWAT contain different populations of stromal cells. The analysis of the adipogenic differentiation capacity of such cell fractions is important for the better understanding of the adipose lineage tracing. However, the relatively low cell numbers in given specific isolates from the SVF generate technical problems, which hamper the efficient study of the differentiation capacity of such cell populations. We asked whether the HFF feeder layer culture technique holds potential to study differentiation of very small cell populations and whether the HFFs' monolayer can be used as an adequate substitute for ASC confluence. To this end, we seeded an increasing number (1000, 5000, 10,000 and 50,000) of ASCs on HFF layer and induced adipogenesis. Oil-Red O staining of differentiated wells revealed that as less as 1000 cells are enough to visualize the differentiation of any potential subset of cells using HFF feeder culture technique (Figure 4(a,b)). In fact, seeding of only 1000 ASCs per well over feeder layer, induction of adipogenic differentiation, staining of the wells with Oil red O and counting of the number of positive cells at d 14 post differentiation showed that 53 ± 5% of the seeded cells differentiated into adipocytes. ASCs were also in the low number sowed on plastic dishes and the sub-confluent cells treated with the adipogenic hormone cocktail. However, under these conditions, the sub-confluent ASCs failed to differentiate into adipocytes (Figure 4c). This highlights the advantage of the HFF monolayer as feeder cells for adipogenic differentiation assays using small progenitor cell populations.

**Figure 4.**
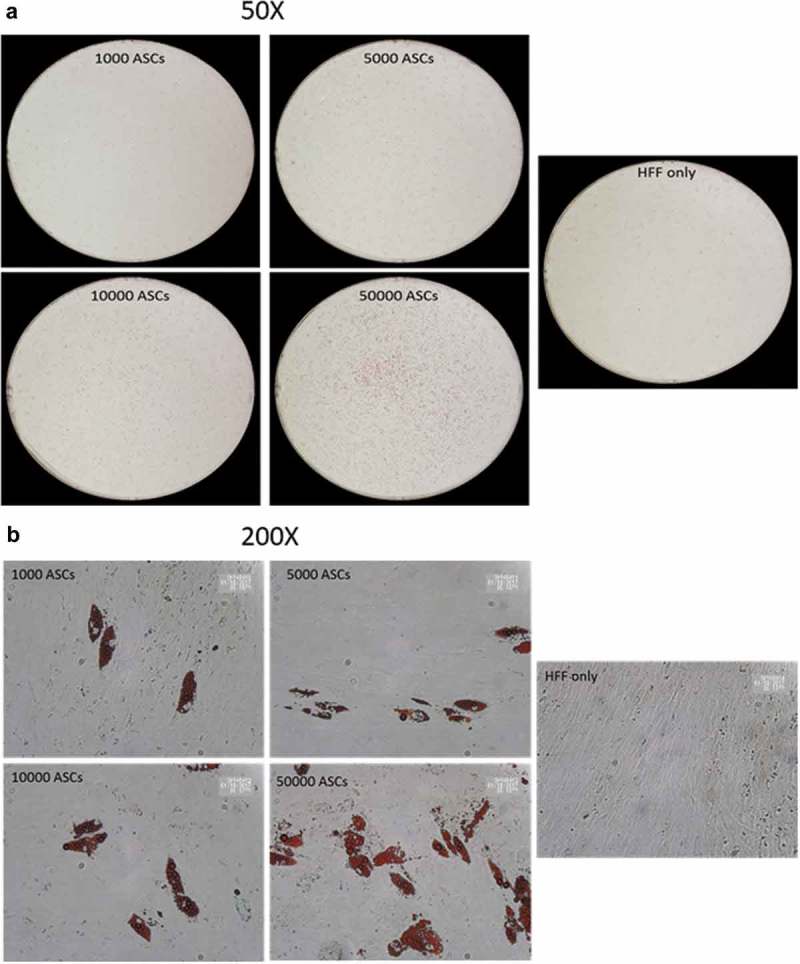
Titration of ASCs number displaying successful differentiation capacity in feeder culture. (a and b) Increasing number of ASCs 1000, 5000, 10,000 and 50,000 were seeded on HFF layer. At d 14 post differentiation, cells were stained with Oil-Red-O and imaged with Nikon camera for whole well (a) or with a light microscope at 200× magnification for details. In (a)and (b), two biological repeats were used each. (c) Increasing number of ASCs 1000, 5000, 10,000 and 50,000 were cultivated independently in pure culture on plastic cell culture dishes. At d 14 post differentiation, cells were stained with Oil-Red-O and imaged with light microscope at 100× magnification. *N* = 3 donors; representative images from one donor are shown.

**Figure 4.**
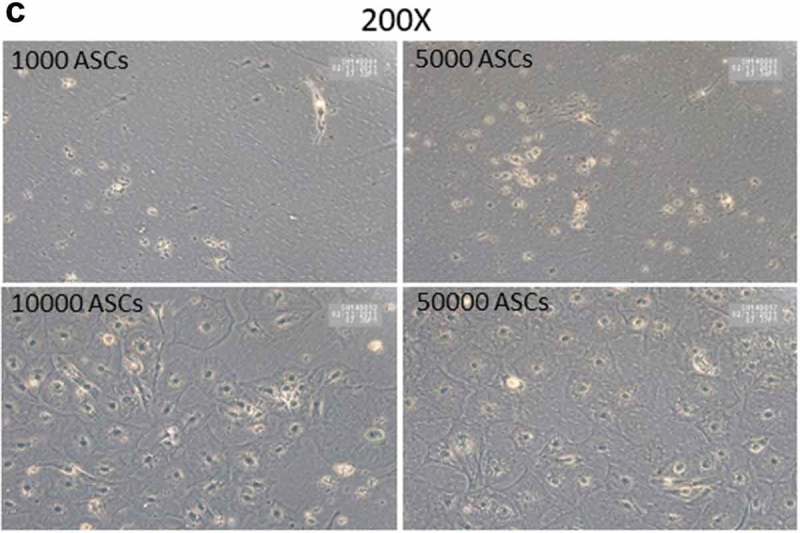
(Continued).

## Discussion

ASCs from the SVF of human sWAT can be isolated by standard procedures and cryopreserved, providing an essential and reliable *in vitro* model system to study mechanisms underlying adipogenesis in primary human adipose progenitor cells *ex vivo* [41]. Extensive *in vitro* expansion of human ASCs is frequently necessary to acquire sufficient numbers of cells for experimental studies; however, this procedure reduces replicative life span and differentiation capacity [35,42,43]. Moreover, the immune-phenotype of ASCs changes during expansion in cell culture on plastic dishes likely due to missing factors from ECM and niche cells and cultivation of the cells in medium with high concentrations of FBS [33–35,44–47]. In fact, it is known that culture conditions and the composition of growth medium are critical for optimal maintenance of the *in vivo* characteristics of ASCs and their proliferative and adipogenic differentiation capacity [6,33,41,43,48].

Good progress has been achieved in the understanding of the impact of cell culture media on adipocyte differentiation of human ASCs [42,43,47,48]. Standard adipogenesis cocktail contains glucocorticoids which activate C/EBPδ, cAMP inducers such as IBMX which activate C/EBPβ expression and insulin which eventually activates PPAR-γ2- and C/EBPα-dependent expression of adipogenic genes [43,49]. Adipogenic differentiation can further be augmented by PPARγ2 agonists, such as thiazolidinediones. However, adipogenic differentiation medium includes also FBS providing a complex mixture of growth factors and mitogens, which contains variable and still undefined factors influencing proliferation, differentiation and survival of adult stem cells [33,34,44–46,50]. While up to 3% FBS increases adipogenic differentiation capacity, FBS levels ≥ 5% have been shown to decrease the differentiation capacity of ASCs likely due to increasing levels of anti-adipogenic factors [6,33,42,43].

Parameters relating to growth medium mainly reflect the impact of soluble endocrine and paracrine factors on adipogenesis. However, the adipose microenvironment contributes also to the direction of stem/progenitor lineage specification [4,13], so that cell shape and ECM would most likely be beneficial for ASC cultivation and differentiation. In the present study, we employed an HFF feeder monolayer as surrogate for niche cells for human ASCs to mimic both biophysical and biochemical effects of the adipose microenvironment. Although fibroblast or stromal feeder layers represent a widely used model system to provide a supportive cellular microenvironment for stem/progenitor cells [51,52], little is known about the employment of fibroblast feeder layers as niche surrogate for human ASCs. HFFs were used for the feeder layer of human ASCs because they are easily obtained from foreskins and, due to their long replicative lifespan, they are highly expandable [39]. It is well known that fibroblasts provide a supportive in vitro environment for many different cell types [53]. This has also been shown for HFFs that support the differentiation of keratinocytes to complex 3D epidermis in epithelial organotypic cultures [54]. We demonstrate in the present study that the HFF feeder strongly supports adipogenic capacity of human ASCs, which were *ex vivo* expanded on a plastic surface to P6. We showed that the efficiency of adipocyte differentiation of these *in vitro* expanded ASCs on the HFF feeder was higher than on plastic surfaces. These data support the notion that specific interactions between the HFF feeder layer and human ASCs exist. Moreover, our findings suggest that moderate *ex vivo* propagation of human ASCs on plastic surfaces is possible, at least to P6, with acceptable preservation of adipogenic capacity when afterwards beneficial micro-environmental conditions are used for adipogenic differentiation. We acknowledge the limitation to our technical approach in using HFFs as surrogate cell shape and ECM rather than using adipose tissue fibroblasts. This needs to be addressed methodologically in further studies of the role of fibroblast feeder layers during adipocyte differentiation. Nevertheless, there is ample evidence for the functional importance of the interaction between ASCs, niche cells and the ECM (reviewed in ref. 4 and 55). For instance, it has been shown that ECM impacts on proliferation and differentiation of ASCs by specific molecular composition and mechanical properties [19,56,57]. Seeding density plays also a positive role for ASC differentiation (Ejaz, Mitterberger-Vogt and Zwerschke, unpublished findings), indicating that cell–cell contacts are important. We hypothesize that under our feeder layer co-culture settings the confluent HFF secretes factors which provide ASCs both a biophysical niche environment and molecular signals essential for optimal differentiation. However, the investigation of mechanistic interaction between foreskin fibroblasts and ASCs is beyond the scope of this study.

An advantage of the feeder layer technique demonstrated in our study is that adipogenesis worked efficiently on HFF feeder even when ASCs are seeded at very low density, suggesting that confluent HFFs provide features that support adipogenesis in ASCs. Although further studies are necessary to support this assumption, our data are in keeping with previous findings suggesting that fibroblast feeder monolayers recapitulate biophysical and biochemical features of niche microenvironment [52]. It is well known that the SVF comprises a mixture of different cell types with different phenotypic signatures and differentiation capacity. The ASC fraction itself represents already a heterogeneous population [7,8,45]. Better exploring the functional characteristics of ASC subpopulations will be important for understanding of the adipose lineage and adipose tissue related metabolic diseases. One hurdle in better characterizing different rare sub-populations in the SVF is a low yield of these cells from cell sorting, which makes it difficult to evaluate these cells in differentiation experiments and other assays *ex vivo* without *in vitro* propagation, which could result in alternation of cell characteristics. Our results indicate that the HFF feeder technique improves adipocyte differentiation capacity and allows to study adipogenesis of human ASCs at low density.
